# Comparison of the myocardial clearance of endothelial progenitor cells injected early versus late into reperfused or sustained occlusion myocardial infarction

**DOI:** 10.1007/s10554-012-0086-5

**Published:** 2012-06-27

**Authors:** Andrea J. Mitchell, Eric Sabondjian, Kimberley J. Blackwood, Jane Sykes, Lela Deans, Qingping Feng, Robert Z. Stodilka, Frank S. Prato, Gerald Wisenberg

**Affiliations:** 1 Department of Medical Biophysics, The University of Western Ontario, London, ON Canada; 2 Department of Medicine, The University of Western Ontario, London, ON Canada; 3 Lawson Health Research Institute, London, ON Canada; 4 Departments of Medicine, Physiology, and Pharmacology, The University of Western Ontario, London, ON Canada; 5 London Health Sciences Centre, University Campus, 339 Windermere Road, London, ON N6A 5A5 Canada

**Keywords:** Myocardial infarction, Stem cell therapy, Residence time, Single photon emission computed tomography

## Abstract

Stem cell transplantation following AMI has shown promise for the repair or reduction of the amount of myocardial injury. There is some evidence that these treatment effects appear to be directly correlated to cell residence time. This study aims to assess the effects of (a) the timing of stem cell injection following myocardial infarction, and (b) flow milieu, on cell residence times at the site of transplantation by comparing three time points (day of infarction, week 1 and week 4–5), and two models of acute myocardial infarction (sustained occlusion or reperfusion). Twenty-one dogs received 2 injections of 30 million endothelial progenitor cells. The first injections were administered by epicardial (n = 8) or endocardial injection (n = 13) either on the day of infarction (n = 15) or at 1 week (n = 6). The second injections were administered by only endocardial injection (n = 18) 4 weeks following the first injection. Cell clearance half-lives were comparable between early and late injections. However, transplants into sustained occlusion infarcts resulted in slower cell clearance 77.1 ± 6.1 (n = 18) versus reperfused 59.4 ± 2.9 h (n = 21) *p* = 0.009. Sustained occlusion infarcts had longer cell retention in comparison to reperfusion whereas the timing of injection did not affect clearance rates. If the potential for myocardial regeneration associated with cell transplantation is, at least in part, linked to cell residence times, then greater benefit may be observed with transplants into infarcts associated with persistent coronary artery occlusion.

## Introduction

Cardiovascular disease remains a major cause of death and disability with its contribution to the world’s burden of disease projected to rise [1]. Acute myocardial infarction (AMI) leads to irreversible myocardial fibrosis, and adverse left ventricular (LV) remodelling. Based on early reports from small animal models [2–6] there has been great interest for cell therapies to a) reduce myocardial injury and b) regenerate new myocardium. Endothelial progenitor cells (EPCs) have been shown to have potential in promoting cardiac repair through angiogenesis [7–10].

Early non-randomized clinical studies suggested beneficial effects on global LV function [11–13] although subsequent trials produced inconsistent results [14–17]. However, recent meta-analyses [18, 19] demonstrated significant, albeit modest, improvements in ejection fraction. Species differences in the absolute size of the infarcts and the nature and timing of the inflammatory response [20] may explain the variation in the degree of improvement between small animal and clinical studies. Amongst other factors, cell residence time has been shown to play an important role in influencing myocardial repair [21]. As well, direct intramyocardial delivery of cells has been explored for both endocardial (catheter based) and epicardial (full or mini-thorocotomy-based) injections with high and equivalent initial cell retention rates [22–25].

The current study was designed to determine the factors that affect cell residence time following direct injection, with infarcts comparable to patients in size and with a similar inflammatory response [20], with clinically applicable and validated imaging tools i.e. cell labelling (Indium-tropolone) and imaging (SPECT) [26, 27], and cell injection techniques (endocardial and epciardial). In this study, we examine cell clearance rates a) on the day of infarction, b) at 1 week and c) 4 weeks following the first injection, in two models of AMI; a sustained occlusion model, and a 2-h occlusion-reperfusion model.

## Methods

### Cell preparation

Autologous EPCs were isolated from the peripheral blood by density gradient centrifugation as described by He et al., and expanded to 30 million cells over 8 weeks, [28]. Cells were labeled with ^111^In-tropolone at 0.1 Bq/cell, a dose not affecting viability or proliferation [29].

### Surgical preparation

Twenty-one 20–24 kg adult female bred-for-research hounds were used. All procedures were approved by the Animal Care Committee of the University of Western Ontario, and performed according to the Guide of the Animal Care and Use of Experimental Animals of the Canadian Council on Animal Care and Use of Laboratory Animals, National Research Council. The results from 14 of these animals, comparing cell retention and clearance rates for endocardial versus epicardial delivery, had previously been reported and the surgical methods described [25]. Anesthesia was induced with propofol and maintained with isoflurane (2 %). Briefly, a myocardial infarction was induced by placing a snare ligature around the left anterior descending coronary artery distal to the first diagonal branch. In 11 animals, the snare was released 2 h following occlusion, and in the remaining 10 animals, the artery was tied off permanently. An additional 3 dogs, 2 with reperfusion, and 1 without, died suddenly within days following infarction, and their clearance times could not be measured.

### Timing of injection

**Fig. 1:**
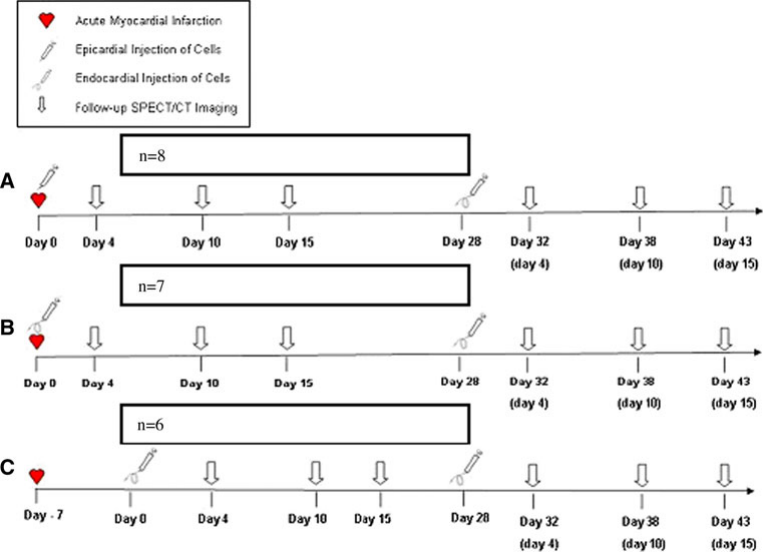
Timing of injection—EPCs labeled with ^111^Indium were injected by **a** subepicardial injection (n = 8) on the day of infarction followed by subendocardial injection at 4 weeks, **b** subendocardial injection (n = 7) on the day of infarction followed by subendocardial injection at 4 weeks, or **c** subendocardial injection at 1 week following infarction (n = 6) followed by subendocardial injection 4 weeks following the first. Follow-up SPECT/CT imaging occurred 4, 10, and 15 days after each cell transplantation. *EPC* endothelial progenitor cell. *SPECT/CT* single photon emission computed tomography/computed tomography

Eight animals received direct epicardial injections at thoracotomy, 4 h following occlusion. The remaining 13 animals received endocardial injections using a LV catheter. Seven of these endocardial injections occurred 4 h after the induction of the myocardial infarction and 6 occurred 1 week later. Eighteen animals received a second endocardial cell injection 4 weeks following the first injection i.e. at 4 or 5 weeks (Fig. 1), as 3 animals died in the interval period, one from congestive heart failure, and the other two from presumed fatal arrhythmias.

### Endocardial injections

Endocardial injections used fluoroscopic guidance with the Stilletto™ Endomyocardial Injection System (Boston Scientific, Natick, MA) as previously described [25]. Briefly, biplane contrast ventriculograms were used to guide the injections into the myocardium, at the periphery of the wall motion abnormalities. Multiple injections (8–10) of 0.1 ml each were delivered using a 26-gauge needle.

### Epicardial injections

At the time of the open thoracotomy, multiple injections (8–10) of 0.1 ml were delivered into the peri-infarct region of the infarction, defined as the peripheral 1 cm of discoloration on the epicardial surface, using a 25-gauge needle [25].

### SPECT/CT imaging and contrast-enhanced CT imaging

Contrast enhanced CT was used to document the sites of injection in relationship to the zone of reduced perfusion. We have previously described the SPECT/CT imaging protocol in detail [25]. After background correction, the ^111^In projection data was reconstructed with three iterations of ordered subset expectation maximization. A volume of interest was defined on day 0 images corresponding to pixels with intensity ≥30 % of the maximum pixel intensity. The number of counts in this volume at each follow-up was used to determine the mean pixel intensity (MATLAB, Mathworks, Natick, MA). The mean pixel intensity in the day 4, 10 and 15 follow-ups were used to create in vivo time-activity curves of the ^111^In activity. They were decay corrected and fit to a monoexponential function in MATLAB and the half-life was reported in hours (Table 1).

**Table 1:** Cell clearance half-lives of endothelial progenitor cells

Canine	Model	Injection 1	Injection 2
		Half-life (hours)	Half-life (hours)
		*Epicardial injection (day 0)*	*Endocardial injection (week 4)*
1	R	48.44	61.18
2		59.70	44.18
3		50.89	52.47
4		84.39	50.26
5	S	40.56	81.84
6		82.62	56.26
7		113.31	*
8		76.27	47.80
		69.5 ± 8.5	56.3 ± 4.7
		*Endocardial injection (day 0)*	
9	R	55.99	47.67
10		68.60	76.43
11		71.25	57.90
12		73.25	60.70
13	S	83.62	66.33
14		90.16	74.27
15		143.9	69.49
		83.8 ± 10.8	64.7 ± 3.8
		*Endocardial injection (week 1)*	
16	R	42.11	69.25
17		40.49	*
18		83.67	48.43
19	S	41.46	*
20		90.50	96.16
21		79.12	53.72
		62.9 ± 9.5	66.9 ± 10.7

Values are mean ± SEM

*R* reperfused myocardial infarction, *S* sustained occlusion myocardial infarction

* SPECT image not acquired

### Statistical analysis

Statistical analysis was performed using SPSS 17.0 (SPSS Inc., Chicago, IL). Comparison between injection techniques was performed using a univariate ANOVA. Comparison between sustained occlusion and reperfused models was performed using a 2-tailed independent samples *t* test. Comparison between first and second injections in each animal was performed using paired sample t-tests. Statistical significance was set at a *P* < 0.05 for all tests. All values are expressed as mean ± SEM.

## Results

### In vitro ^111^In-tropolone labeling

In 39 cell labeling procedures, the average labeling efficiency was 77.1 ± 1.5 %, resulting in an average dose of radioactivity delivered per cell of 0.1 ± 0.003 Bq/cell.

### Cell delivery efficiency

After each injection, the syringe and/or catheter used for the injection were evaluated for the amount of retained activity. For the first injection 8.4 ± 1.3 % of the activity remained in the syringe after the epicardial injections, while 15.7 ± 2.1 % of the activity remained in the syringe and catheter after the endocardial injections (*p* = 0.02).

The ratio of activity in the heart over the total activity in the body i.e. the myocardial retained activity, was similar between the epicardial (56.7 ± 6.0 %) and endocardial (59.5 ± 5.5 %) injections (*p* = 0.76).

### SPECT imaging

#### Epicardial versus endocardial injection (day 0) Table 1

The clearance half-lives for cells injected into the epicardium were not significantly different from cells injected into the endocardium on the day of the infarction (*p* = 0.313).

#### Endocardial injections: day 0 versus week 1

The clearance half-lives of the cells injected into the endocardium on the day of infarction were similar to the clearance half-lives of cells injected into the endocardium 1 week after the infarction (*p* = 0.185).

#### Timing of injection (0–1 week vs. 4–5 weeks)

All animals received two cell injections, the first either on the day of infarction or 1 week later, and the second 4 weeks following the first. Analysis was performed on the clearance half-lives between the first and second injection in each of the three categories of first injection (epicardial injection on day 0, endocardial injection on day 0 and endocardial injection at week 1), and separately grouping all the early injections and comparing them to the late (4–5 week) injections. Although the clearance half lives comparing the smaller groups’ early versus late injections did not approach statistical significance, when comparing all the early injections (Day 0 and Week 1) versus late injections, there was a trend towards a difference for the first (n = 18, 73.6 ± 5.6 h) versus second (n = 18; 61.9 ± 3.3 h) injections (*p* = 0.074).

#### Sustained occlusion versus reperfused tissue

A comparison between sustained occlusion (n = 10) and reperfused (n = 11) models for injection 1 revealed that the cell clearance half-lives of 84.2 ± 9.6 and 61.6 ± 4.7 h, for the occlusion and reperfused models respectively, were significantly different (*p* = 0.042), but no significant difference for injection 2, 68.2 ± 5.0 h versus 55.5 ± 3.3 h (*p* = 0.086) (Table 2). However, when grouping all the sustained occlusion versus all the reperfused injections, there was a significant difference for sustained occlusion 77.1 ± 6.05 (n = 18) versus reperfused 59.4 ± 2.9 h (n = 21), *p* = 0.009.

**Table 2:** Mean cell clearance half lives of endothelial progenitor cells in sustained occlusion and reperfused models of myocardial infarction

n	Model	Injection 1	Injection 2
		Half-life (hours)	Half-life (hours)
11	R	61.6 ± 4.7^a^	55.5 ± 3.3
10	S	84.2 ± 9.6	68.2 ± 5.0

Values are mean ± SEM

*R* reperfused myocardial infarction, *S* sustained occlusion myocardial infarction, *n* sample size

^a^R versus S *p* = 0.042

#### Contrast enhanced CT

**Fig. 2:**
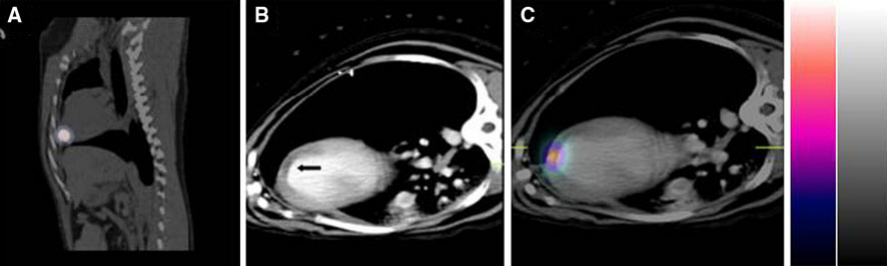
SPECT/CT—day 0 imaging of ^111^Indium labeled EPCs transplanted in a canine heart by endocardial injection **a** Coronal fused SPECT/CT image showing activity localized to the heart. **b** Transaxial contrast-enhanced CT image with arrow denoting hypo-enhanced region within the infarction, **c** Transaxial contrast-enhanced CT image fused with SPECT image of transplanted EPCs. *EPC* endothelial progenitor cell, *SPECT/CT* single photon emission computed tomography/computed tomography

The post-contrast enhanced cardiac SPECT/CT showed areas of hypo-perfusion in all animals indicating low blood flow. All injections were localized within the zone of reduced contrast towards the periphery of this zone (Fig. 2).

## Discussion

Our study is the first to assess some of the factors that affect cell residence time in a large animal model of myocardial infarction. We have shown that endocardial and epicardial injections have similar residence times and may direct further research towards endocardial injections as they are technically easier to perform clinically, although safety issues may be a concern. Regarding the other tissue based parameters, previous large animal studies tracked cells under a single condition, i.e. single time of injection or sustained occlusion/reperfusion, but not both, which is unique to our study. Our laboratory has previously validated the technique of Indium tropolone labeling [26, 27], for quantitative measurement of cell clearance. There are other methods to track cells following transplantation [30–39], some of which have demonstrated approximately the same range of clearance half-times seen in our study [38].

However, the status of the tissue, and cell type appear to have a significant bearing on clearance rates, based on work in a rat model using optical imaging [40]. Longer clearance times for stromal cells were found if they were (a) injected at 30 min versus 5 days, (b) transplanted into normal versus infarcted myocardium, (c) syngeneic transplants versus allogeneic and (d) treated with immunosuppressive therapy.

Also, the time of injection appears to have an effect as Schachinger reported that cell retention declined as the time elapsed since the infarct increased [41] in a clinical study of patients ranging from 5 days to 17 years.

### Endocardial injections: day 0 versus week 1

Macrophage invasion in canine myocardial infarction has a slow sustained progression starting at 24 h, and peaking at 7 days [20]. In our study, the endocardial injections on day 0 occurred 4–5 h after coronary occlusion, before the major infiltration of macrophages, whereas the injection at 1 week occurred at its peak. Had the radioactive debris from dead transplanted cells been engulfed by the resident macrophages, we would have observed an apparent prolonged cell clearance at 1 week. However, we found similar clearance half-lives at day 0 and week 1 arguing against this hypothesis.

### Timing of injection (0–1 weeks vs. 4 weeks)

Each animal received two cell injections, separated by 4 weeks, with a trend towards longer half-lives with the earlier injections. This may have been related to lower blood flows initially with a gradual increase over time related to augmented collateral supply. However, we did not measure regional myocardial perfusion to validate this hypothesis. In theory, earlier cell injections may be more beneficial in limiting the full evolution of an infarct, but this is also unproven. Further, providing an adequate number of cells to culture expand and then transplant for early injections at 1 week would be problematic, and their safety remains a concern.

### Sustained occlusion versus reperfused tissue

The residence time following transplantation into the sustained animals was longer by 17.7 h on average compared to reperfused animals. This difference would produce approximately twice as many cells still resident at the site of infarction at 10 days after injection (1/8th vs. 1/16th). If treatment effect is correlated with cell residence times, this would suggest that greater myocardial regeneration would occur, everything else being equal. However, we did not assess the extent of infarction or alternatively, the viability of the infarcted/ischemic tissue in this study to make this direct link.

### Variation in cell clearance

These may be related to differences in infarct size, variations in collateral flow around the coronary artery occlusion, in basal heart rate, and blood pressure between animals. Also, the injections were targeted solely based on wall motion abnormalities, which could be caused by stunned, hibernating, or infarcted myocardium (viability status was not used to direct the injections). The contrast enhanced CT images confirmed that, in all cases, the injections had been placed within the zone of reduced perfusion, and generally towards its periphery.

### Safety and clinical relevance

Here, we have shown that careful experiments using a large animal model (canine) were undertaken with a relatively low overall attrition rate of 6/24 (25 %), primarily from arrhythmias, which is consistent with our experience over the past 25 years with this model. There were no perforations. Gyongyosi has reported on the safety of early post infarction injections in 60 patients receiving direct myocardial injections at either 3–6 weeks or 3–4 months 42], and Krause demonstrated the safety of direct endocardial injections an average of 10.5 days after infarction [43], with no peri-procedural complications. We have shown here that late injections at 4–5 weeks are associated with comparable cell clearance as for injections within the first week.

### Study limitations

The focus of this study was to determine if clinically significant differences in transplanted cell residency time were a function of infarct status (reperfused vs. chronic), time of injection after infarct event or route of cell administration (epicardial vs. endocardial). Hence protocol design was optimized to achieve these results with a minimum number of dogs. This was achieved by injecting each animal twice (day 0 or 1 week followed by a second injection 4 weeks after the first). This allowed post hoc analysis with paired sample t-testing for increased statistical power for the same number of animals but also introduced study limitations. Treatment effect could not be investigated as 4 weeks is, in our experience [44], too short a time after the first injection to assess this. Additionally, with two injections, any treatment effect at a remote time point e.g. 12 weeks could not be uniquely attributable to one of the two injections. Another concern is the potential contaminating effect of any residual treatment response or inflammatory reaction, of the first injection on the 4–5 week residence times. However, on review of the data, (Table 1), there was no consistent pattern seen from first to second injection. The relatively small sample sizes in each group limited the size of the effects that can be seen with statistical certainty. For example we would have needed much larger sample sizes (27–62 animals/group based on Cohen’s effect size for two independent means, *p* < 0.05, power = 0.80) to have demonstrated that the differences between injection 1 and injection 2 were significant or the differences between the epicardial versus endocardial injections were different. However the aim of the study was to determine if differences were large and hence would likely have an impact in clinical application and this, we argue, was achieved.

## Conclusions

The transplanted cell clearance kinetics injected on the day of infarction, at 1 week and 4–5 weeks following infarction showed no statistical difference, although there was a trend to longer half-lives for the earlier injections.

Sustained occlusion infarcts, which generally are larger than reperfused ones, and would benefit most from regeneration, had statistically longer cell retention times. Although it is beyond the scope of this article to discuss in detail, the current clinical trial evidence suggests no prognostic benefit to delayed re-opening of a chronically occluded coronary artery [45]. If cell residence time is associated with treatment effect size and/or transplanted cell proliferation, relatively greater effects would be observed following transplantation into the peri-infarct region of sustained occlusion infarcts.

## Abbreviations

AMI: Acute myocardial infarction
CT: Computed tomography
EPC: Endothelial progenitor cell
LV: Left ventricular
SEM: Standard error of the mean
SPECT: Single photon emission computed tomography
